# Clinical impact of sarcopenia assessment in patients with hepatocellular carcinoma undergoing treatments

**DOI:** 10.1007/s00535-020-01711-w

**Published:** 2020-08-03

**Authors:** Giovanni Marasco, Matteo Serenari, Matteo Renzulli, Luigina Vanessa Alemanni, Benedetta Rossini, Irene Pettinari, Elton Dajti, Federico Ravaioli, Rita Golfieri, Matteo Cescon, Davide Festi, Antonio Colecchia

**Affiliations:** 1 Department of Medical and Surgical Sciences, University of Bologna, Via Massarenti 9, 40126 Bologna, Italy; 2 Radiology Unit, Sant’Orsola Malpighi Hospital, Via Albertoni 4, 40138 Bologna, Italy; 3 Gastroenterology Unit, University Hospital Borgo Trento, Verona, Italy

**Keywords:** Sarcopenia, Hepatocellular carcinoma, Liver resection, Sorafenib

## Abstract

Changes in body composition are associated with poor outcomes in cancer patients including hepatocellular carcinoma (HCC). Sarcopenia, defined as the loss of skeletal muscle mass, quality and function, has been associated with a higher rate of complications and recurrences in patients with cirrhosis and HCC. The assessment of patient general status before HCC treatment, including the presence of sarcopenia, is a key-point for achieving therapy tolerability and to avoid short- and long-term complications leading to poor patients’ survival. Thus, we aimed to review the current literature evaluating the role of sarcopenia assessment related to HCC treatments and to critically provide the clinicians with the most recent and valuable evidence. As a result, sarcopenia can be predictive of poor outcomes in patients undergoing liver resection, transplantation and systemic therapies, offering the chance to clinicians to improve the muscular status of these patients, especially those with high-grade sarcopenia at high risk of mortality. Further studies are needed to clarify the predictive value of sarcopenia in other HCC treatment settings and to evaluate its role as an additional staging tool for identifying the most appropriate treatment. Besides, interventional studies aiming at increasing the skeletal muscle mass for reducing complications and increasing the survival in patients with HCC are needed.

## Introduction

Hepatocellular carcinoma (HCC) is one of the most common malignancies worldwide [1]. The identification of patients with a high-mortality risk is the key-point in the choice of the most adequate treatment for each patient with HCC, according to the patient’s specific prognosis. Thus, several prognostic staging systems have been developed, such as Barcelona Clinic Liver Cancer (BCLC) and others [1]. Nevertheless, its high-mortality rate, the prognostic factors for HCC remain controversial since the long-term prognosis of HCC is associated with several factors, mainly represented by the liver functional reserve and the stage of cancer progression [1, 2]. In the past decade, the performance status developed by the Eastern Cooperative Oncology Group (ECOG) was added to the BCLC staging system in order to provide a parameter for the general assessment of patients status, ranging from fully active to dead [1], the performance status has been previously associated with both tumoral and cirrhotic factors and accurately predicts long‐term survival in HCC patients [3]. However, to date, the available staging and prognostic systems do not include other parameters for assessing the general performance and nutritional and functional status of the patient with HCC. Interestingly, in addition to these well-known factors, previous studies demonstrated that changes in body composition are associated with poor outcomes in cancer patients including HCC [4]. Indeed, a progressive and generalized skeletal muscle disorder, defined as sarcopenia, is associated with an increased likelihood of adverse outcomes including falls, fractures, physical disability and mortality [1, 2]. More in deep, sarcopenia is characterized by the loss of skeletal muscle mass, quality and strength [5]. During the past decades, several methods have been proposed to assess sarcopenia (Table 1). This disorder can be due to aging (primary sarcopenia) or acute and chronic illness (secondary sarcopenia), including chronic liver diseases [4]. Moreover, it has been associated with poor prognosis in a lot of malignancies as pancreatic cancer [6], colorectal liver metastases [7], melanoma [8], lung cancer [9] and esophageal neoplasia [10] and significantly increases morbidity and mortality after surgery for cancer [11], other than being associated with the outcome of patients with HCC [12].

**Table 1:** Commonly used methods for assessing sarcopenia

Methods	How to	Units	Cut-offs	Pro	Cons
HGS	Measured using a hand dynamometer. The highest values for both right and left handgrip strength from two measurements were averaged, and then used for analysis	kg	M: < 27 kg W: < 16 kg M: < 30 kg W: < 15 kg	Validated cut-off; Simple and inexpensive	Not representative of overall sarcopenia
PMI	Total bilateral psoas muscle area at the middle of the third lumbar vertebra (L3) level (cm^2^), shown by CT, and height (m)	cm^2^/m^2^	M < 5.37 cm^2^/m^2^, W: < 3.4 cm^2^/m^2^ M: < 6.36 cm^2^/m^2^ W: < 3.92 cm^2^/m^2^	Simple and commonly used	Not representative of overall sarcopenia
TPV	Total psoas volume of the right psoas muscle was calculated semi‐automatically, by manual outlining of the boarders of the muscle, shown by CT, starting at the level of the last thoracic or first lumbar vertebra continuing until the psoas muscle becomes indistinguishable from the iliopsoas muscle	cm^3^	M: < 194.9 cm W: < 99.2 cm	Easy to calculate	Not representative of overall sarcopenia
PMTH	Psoas mass thickness, measured on CT at the level of the umbilicus, or at L3 or L4 was normalized by division by height	mm/m	16.8 mm/m at umbilicus level 14 mm/m al L4 level	Easy to calculate	Different level evaluated (L3, L4, umbilicus) Not representative of overall sarcopenia
TPMT	TPMT-L3: defined as the transversal diameter of the right psoas muscle perpendicular to the largest axial psoas muscle diameter at the L3 endplate, measured on CT. The results were normalized to body height TPMT‐umbilical: defined as the transversal diameter of the right psoas muscle perpendicular to the largest axial psoas muscle diameter at the level of the umbilicus. Results were normalized to body height	mm/m	M: < 10.7 mm/m W: < 7.8 mm/m	Easy to calculate	Different level evaluated The level of umbilicus could be influenced from ascites Not representative of overall sarcopenia
PSMI	Bilateral, total paraspinal muscle area (psoas major and minor muscles, quadratus lumborum muscles, transvers spinal muscles and erector spinae muscles) at the L3 endplate, measured on CT. The results were normalized by height	cm^2^/m^2^	M: < 26.3 cm^2^/m^2^ W: < 20.8 cm^2^/m^2^	CT images of a specific lumbar vertebral landmark (L3) correlated significantly with whole-body muscle	Not representative of overall sarcopenia
SMA	Assessed as the mean density (HU) of the entire measured cross-sectional muscle area at L3, measured on CT	HU	–	Reflect both to micro- and macroscopic changes in muscle architecture and composition	There is no universal consensus on this method for routine clinical practice
SMI	Skeletal muscles at the L3 or L4 level included the erector spinae, transverse abdominis, psoas, quadratus lumborum, internal and external oblique abdominal muscle and the rectus abdominis muscle, measured on CT, normalized for patient height	cm^2^/m^2^	L3 level: M: < 36.2 cm^2^/m^2^ W: ≤ 29.6 cm^2^/m^2^ M: < 52.4 cm^2^/m^2^ W: < 38.5 cm^2^/m^2^ W: < 41 cm^2^/m^2^ M: < 53 cm^2^/m^2^ With BMI > 25 and < 43 cm^2^/m^2^ with BMI > 25 L4 level: < 52.4 cm^2^/m^2^	Most used CT based technique Precise measures of body composition	Different cut-offs
SMI by BIA	Appendicular SMM/height squared by BIA	kg/m^2^	M: < 7.0 kg/m^2^ W: < 5.5 kg/m^2^	BIA equipment is affordable, widely available and portable	BIA measurements can also be influenced by hydration status
MAMC	MAMC (cm) = MAC—(0.314 × TSF [mm])	cm	–	Easy to calculate; Simple and inexpensive	Not representative of overall sarcopenia
TSF	Measured by one experienced observer with caliper at the middle point between the acromion and the olecranon of the non-dominant arm	cm	–	Bedside technique Simple and inexpensive	Not representative of overall sarcopenia
LBM	0.306x[skeletal muscle at L3 using CT (cm2)] + 6.06	kg	–	CT images of a specific lumbar vertebral landmark (L3) correlated significantly with whole-body muscle	Not representative of overall sarcopenia
US- PTHR	Mean of psoas diameter divided, measured on US, by patient’s height	mm/m	–	US-based technique Assess both muscle quantity and quality	No valid cut-off
US-PMI	Psoas radius square, measured on US, divided by patient’s height square	cm^2^/m^2^	–	US-based technique Assess both muscle quantity and quality	No valid cut-off

*BIA* Bioelectrical impedance analysis, *HGS* handgrip strength, *MAC* Midarm circumference, *MAMC* midarm muscle circumference, *PSMI* Paraspinal muscle index, *PMI* psoas muscle index, *SMA* skeletal muscle attenuation, *SMI* skeletal muscle index, *SMI* skeletal muscle index, *SMM* skeletal muscle mass, *TPV* Total psoas volume, *LBM* Total lean body mass, *TSF* triceps skinfold thickness, *TPMT* Transversal psoas muscle thickness, *PMTH* psoas muscle thickness by height, *US- PTHR* Ultrasound Psoas to height ratio, *US-PMI* Ultrasound Psoas muscle index

In fact, sarcopenia has been associated with a higher rate of complications and recurrences in patients with cirrhosis and HCC undergoing resection [13]. While few studies exist on the prognostic role of sarcopenia after ablative treatments [14], such as radiofrequency ablation, chemoembolization and radioembolization, a growing number of studies have been conducted in patients undergoing systemic therapies for HCC, such as the multi-kinase inhibitor Sorafenib [15, 16]. In this latter setting, where the assessment of the general status of the patient is crucial for achieving therapy tolerability, sarcopenia was an independent predictor of poor survival, scarce tolerance to chemotherapy and higher toxicity in these patients [15, 16].

Therefore, the aim of this review was to critically revise the available evidence on the role of sarcopenia assessment in all HCC treatment settings, in order to assess whether it can be considered a reliable tool for stratifying patients’ prognosis before treatment and to address further research into the field.

We conducted a Medline and PubMed search from inception to December 2019 using the search terms ‘sarcopenia’, ‘muscle’, ‘body composition’, ‘hepatocellular carcinoma’, followed by a manual review of the literature to select articles evaluating the influence of sarcopenia on HCC treatments and outcomes.

## Curative treatments

### Liver resection

**Fig. 1:**
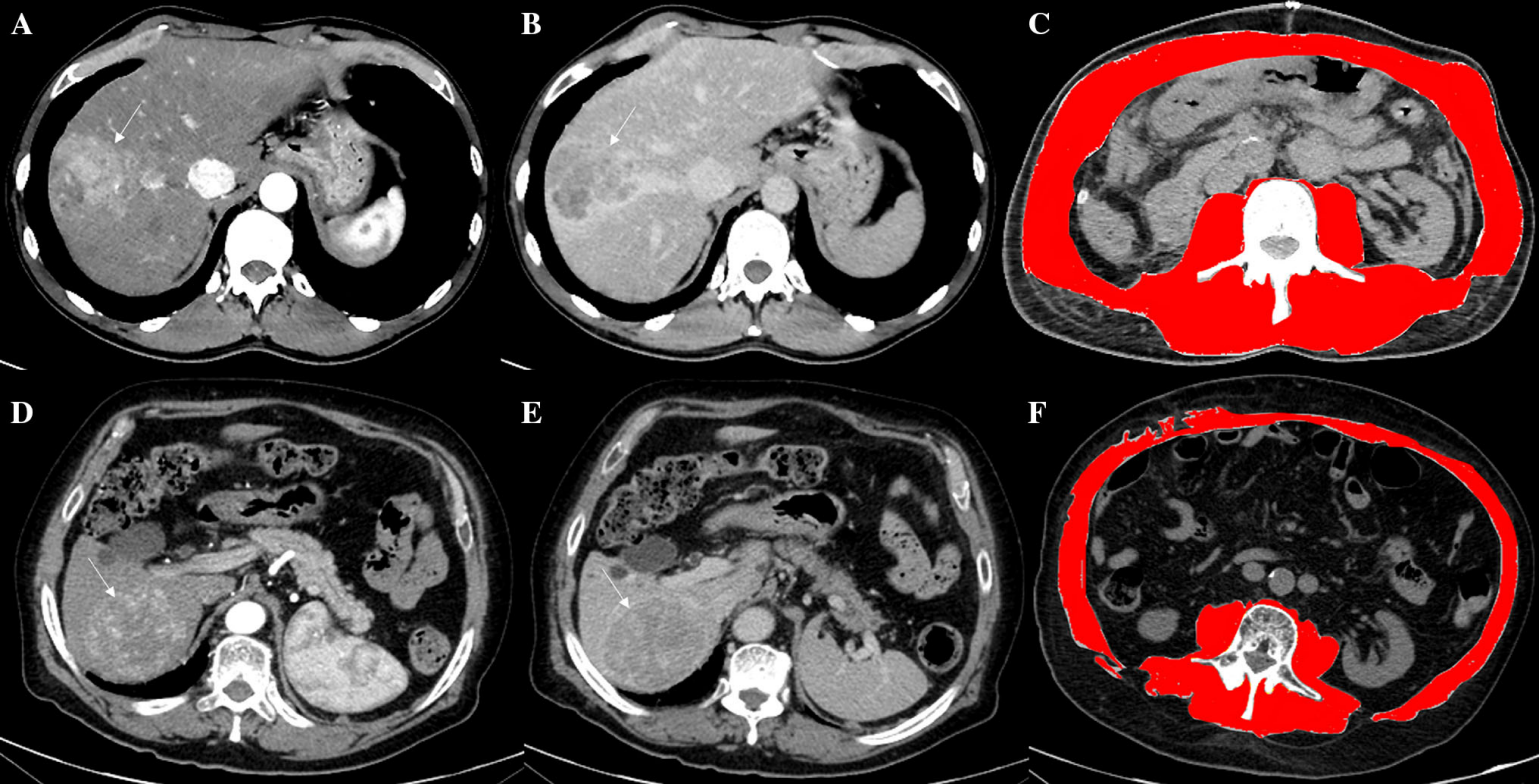
The Computed Tomography images of two different patients (fist: **a**, **b**, **c**; second: **d**, **e**, **f**) demonstrated two large hepatic lesions consistent with hepatocellular carcinoma due to the arterialization (arrows in **a** and **d**) coupled with wash-out of contrast media in the delayed phases (arrows in **b** and **e**). The diagnosis was confirmed by histology after surgical treatments in both patients. The evaluations at the level of the soma of the third lumbar vertebra by using dedicated free software revealed no sarcopenia in the first patient (**c**) and sarcopenia in the second one (**f**)

The usefulness of the assessment of the nutritional status in patients undergoing hepatectomy is known since 1994, when Fan et al. [17] studied two groups of patients randomly assigned to receive or not preoperative intravenous nutritional support (branched-chain amino acids, lipid emulsion and dextrose). The main difference found was a reduction in septic complication after surgery, the need of diuretic therapy to reduce ascites and less deterioration of liver function as measured by the change in the rate of clearance of indocyanine green ( – 2.8% vs.  – 4.8% at 20 min, *p* = 0.05), and more importantly a reduction of in-hospital mortality. As regards studies specifically addressing the role of sarcopenia in patients undergoing liver resection (summarized in Table 2), the first study evaluating the relationship between sarcopenia and the prognosis of patients with HCC following hepatic resection was made by Harimoto et al. in 2013 [18]. In this study, the cross-sectional areas of skeletal muscles (psoas, erector spinae, quadratus lumborum, rectus and transversus abdominis) in L3 region were normalized for height (cm^2^/m^2^) [18]. The cut-off values used for skeletal muscle associated with overall survival (OS) were defined, respectively, as 43,75 cm^2^/m^2^ for men and 41,10 cm^2^/m^2^ for women [7]. This study [18] concluded that sarcopenia was not associated with age, whereas it was significantly correlated with liver dysfunction as indicated by abnormal serum albumin levels and indocyanine green retention test (ICGR-15) values, as well as with reduced body mass index (BMI) values. Patients with sarcopenia showed a significantly impaired prognosis than those without, for overall (*p* = 0,001) and recurrence-free survival (*p* = 0,013). The first European study which evaluated the impact of sarcopenia on hepatectomy was reported by Voron et al. [20] in 2015. In this study, sarcopenia was defined as skeletal muscle index (SMI) (example in Fig. 1) less than 52.4 cm^2^/m^2^ for men and less than 38.9 cm^2^/m^2^ for women. Sarcopenia was correlated with the presence of a more undifferentiated HCC (*p* = 0.015) and the presence of satellite nodules (*p* = 0.031) than non-sarcopenic patients. Voron and collaborators [20] also showed that sarcopenia was a strong and independent prognostic factor for mortality (Hazard Ratio [HR] = 3.19, 95% Confidence intervals [CI] 1.28–7.96; *p* = 0.013) and recurrence (HR = 3.03, 95% CI 1.67–5.49; *p* = 0.001) after liver resection for HCC. These results were confirmed by a later study by Takagi et al. [22], which used a different cut-off to define sarcopenia, respectively, 46.4 cm^2^/m^2^ for men and 37.6 cm^2^/m^2^ for women, but they also found the overall 5-year survival rate after hepatectomy was significantly lower in the sarcopenic group compared to the non-sarcopenic group (58.2% vs. 82.4%, log-rank *p* = 0.0002) [22], moreover, they found that sarcopenia was correlated with the presence of microvascular invasions (*p* = 0.003) and the tumor stage (*p* = 0.015) [22].

**Table 2:** Studies assessing sarcopenia in patients undergoing liver resection

Author (year)	Region	N. patients	Outcome	Methods for sarcopenia assessment	N. of sarcopenic patients	Cut off
Fan (1994) [17]	Asia	124 (64 nutritional support group vs 60 control)	Complications after surgery	Midarm circumference Triceps skin-fold thickness HGS	–	–
Harimoto (2013) [18]	Asia	186	OS Recurrence	L3-SMI at CT	75	M: < 43,75 cm^2^/m^2^ W: < 41,10 cm^2^/m^2^
Dello (2013) [19]	Europe	40	TFLV	L3-SMI at CT	27	M: < 55.4 cm^2^/m^2^ W: < 38.9 cm^2^/m^2^
Voron (2015) [20]	Europe	109	Mortality Recurrence	L3-SMI at CT	59	M: < 52.4 cm^2^/m^2^ W: < 38.9 cm^2^/m^2^
Otsuji (2015) [21]	Asia	256	Hospital stay Complications after surgery PHLF	TPA/ height	85	M: < 536 cm^2^/m^2^ W: < 378 cm^2^/m^2^
Takagi (2016) [22]	Asia	254	5-year OS	L3-SMI at CT	118	M: < 46.4 cm^2^/m^2^ W: < 37.6 cm^2^/m^2^
Yabusaki (2016) [23]	Asia	195	Recurrence	L3-SMI at CT	89	M: < 43,75 cm^2^/m^2^ W: < 41,10 cm^2^/m^2^
Hamaguchi (2019) [24]	Asia	606	Mortality Recurrence	VSR L3-SMI L3-IMAC	–	VSR M: < 1.325 W: < 0.710 SMI M: < 40.31 cm^2^/m^2^ W: < 30.88 cm^2^/m^2^ IMAC M: < -0.358 HU W: < -0.229 HU
Kobayashi (2019) [25]	Asia	465	Mortality Recurrence	L3-SMI visceral adipose tissue area	Sarcopenic non- obesity = 31 Sarcopenic obesity = 31	M: < 40.31 cm^2^/m^2^ W: < 30.88 cm^2^/m^2^ > 100 cm^2^

*N*. Number, *HGS* Handgrip strength, *OS* Overall Survival, *L3* third lumbar vertebra, *SMI* cross-sectional areas of skeletal muscle (cm^2^)/patient’s height (m^2^), *CT* computed tomography, *M* male, *W* women, *TFLV* total functional liver volume, *TPA* total psoas muscle area, *PHLF* post-hepatectomy liver failure, *VSR* visceral adipose tissue area (cm^2^)/sub- cutaneous adipose tissue area (cm^2^), CT attenuation value of the multifidus muscles (HU)/CT attenuation value of the subcutaneous fat (HU) (IMAC)

Interestingly, the risk of recurrence after liver resection was found to be associated with the combined presence of a sarcopenic high BMI. Indeed, another research group [23], using further different cut-off values for SMI (43.75 cm^2^/m^2^ and 41.10 cm^2^/m^2^ for males and females, respectively), found that there was no difference in the incidence of post-operative complications and 90-day mortality between the patients with and without sarcopenia; however, they further stratified their cohort according to the BMI (BMI < 22 and BMI > 22), finding at multivariate analysis that poorly differentiated tumor cells, microvascular invasion and a low SMI were independently associated with an increased risk of recurrence (HR = 1.6; 95% CI, 1.1–2.5; *p=* 0.02).

As regards the immediate outcomes after liver resection, sarcopenic patients were found to have a smaller preoperative total functional liver volume (TFLV) compared with non-sarcopenic patients undergoing liver resection (2% vs 2.3%, *p* < 0,05) [19], and these findings may negatively affect short-term outcomes after liver resection. However, little is known about the effect of sarcopenia on short-term outcome after resection, such as the occurrence of post-hepatectomy liver failure,in a Japanese study [21] evaluating the impact of total psoas muscle area (TPA), patients with sarcopenia revealed a significantly higher rate of liver failure (International study group of liver surgery grading [ISGLS] grade C and B) (33% vs 16%, *p* = 0.003), major complications assessed with Clavien grade C3 (54 vs 37%, *p* = 0.011), and intra-abdominal abscess (29 vs 18%, *p* = 0.040) and a longer hospitalization (39 vs 30 days, *p* < 0.001) than those without sarcopenia. The optimal cut-off for normalized TPA [21] associated with the development of liver failure was for males 567 mm^2^/m^2^, and for females 395 mm^2^/m^2^, respectively. The odds ratio (OR) reported for post hepatectomy liver failure (PHLF) prediction by sarcopenia was 2.44 (95% CI 1.20–4.99, *p* = 0.012). More recent studies [24, 25] showed the role of sarcopenic obesity on prognosis after hepatectomy; in the study by Kobayashi et al. [25] patients were classified on the basis of body composition into four groups as non-sarcopenic non-obesity (39%), non-sarcopenic obesity (47%), sarcopenic non- obesity (7%) and sarcopenic obesity (7%). The OS and the recurrence-free survival rates after hepatectomy for HCC were significantly lower in the sarcopenic obesity group than in the non-sarcopenic non-obese group (*p* = 0.002 and *p* = 0.003, respectively). In conclusion, the preventive study of sarcopenia before liver resection spreads only over the past decade and with different methods and proposed cut-offs; the depletion of muscle mass in HCC patients seems to be dependent on the underlying liver disease and to the aggressiveness of the HCC. While there are only isolated studies that need validation regarding the risk of HCC recurrence after treatment and PHLF, robust data are available on the predictive role of sarcopenia assessment before liver resection and patients’ OS.

### Radiofrequency ablation therapy

To date, poor data exist about the impact of sarcopenia on radiofrequency ablation therapy (RFA). Only one study [26] evaluated the predictive role of skeletal muscle mass on survival for HCC patients undergoing percutaneous RFA therapy. In this study, the evaluation for muscle mass was conducted using pre-treatment psoas muscle index (PMI, cm^2^/m^2^) on the Computed Tomography (CT) images. Using Receiver Operating Characteristics (ROC) analysis for survival, the optimal cut-off point for PMI was 6.31 cm^2^/m^2^ in males (Area under ROC curve [AUROC] 0.74, sensitivity 86.5%, specificity 56.8%) and 3.91 cm^2^/m^2^ in females (AUROC 0.76, sensitivity 72.0%, specificity 78.3%). Patients with low PMI showed a reduced rate of survival than those without, being PMI an independent predictor for survival (HR 6.867 at multivariate analysis) [26]. Even less is known about the role of sarcopenia on percutaneous ethanol injection, so it might be interesting to evaluate its impact also on the outcome of this percutaneous technique.

### Liver transplantation

Sarcopenia is frequently encountered in patients with End-Stage Liver Disease (ESLD), and, for this reason, several studies have tried to show that sarcopenia, in turn, could be a major predictor of adverse clinical outcome measures also in liver transplantation (LT) [27](Table 3). Notably, most of the studies evaluating the prognostic role of sarcopenia in LT setting included patients undergoing or who have undergone LT irrespective of the presence of HCC. In addition, different skeletal muscle mass measurements have been used or even functional assessments of strength/performance, thus making comparisons among different studies difficult to perform. Regardless of the various definitions of sarcopenia used, several studies have demonstrated that it represents a risk factor for mortality among LT candidates. In particular, sarcopenia measured by lumbar three skeletal muscle index (L3 SMI) was first demonstrated by Tandon et al. [45] to be a 2.4-fold independent risk factor for mortality in patients awaiting LT. Similarly, in another study, transverse psoas muscle thickness (TMPT)/height ratio was associated with an increase of 15% of waiting list mortality for every unit of decrease of TMPT [31]. Even larger and multicenter studies confirmed the correlation between sarcopenia and waiting list survival [38]. Such findings prompted the transplant community to add sarcopenia to the Model for End-stage Liver Disease (MELD) score for better predicting patients’ survival while in-list. In fact, although MELD score has always been used to prioritize patients with ESLD for LT, it may underestimate disease severity in many cases. Montano-Loza et al. [34] described how MELD-sarcopenia (defined as L3 SMI) score was associated with improvement in the prediction of 3-month mortality in patients evaluated for LT, with the best benefit in patients with low MELD score (< 15). In particular, if present, sarcopenia (i.e. males with BMI < 25: < 43 cm^2^/m^2^, males with BMI ≥ 25: < 53 cm^2^/m^2^, females: 41 cm^2^/m^2^) was equivalent to adding 10 points to MELD score. On the contrary, Van Vugt et al. [40] in a multicenter European cohort of 585 patients listed for LT, showed that although the presence of sarcopenia was associated with an increased waiting list mortality, adding these measurements to the currently used organ allocation system in the Netherlands did not provide any additional benefit in predicting mortality compared to MELD score alone [40].

**Table 3:** Studies assessing sarcopenia in patients undergoing liver transplantation

Author (year)	Region	N. patients (N. HCC patients)	Outcome	Methods for sarcopenia assessment	N. of sarcopenic patients	Cut off
Krell (2013) [28]	America	207 (52)	Infections	TPA	–	–
Di Martini (2013) [29]	America	338 (NA)	Hospital stay Intensive unit stay	SMI	68	M: < 53.4 cm^2^/m^2^ W: < 38.5 cm^2^/m^2^
Kaido (2013) [30]	Asia	124 (39)	Survival after LT	Skeletal muscle mass by BIA	47	< 90% of the standard
Durand (2014) [31]	Europe	562 (258)	Mortality	TPMT/height	–	–
Lee (2014) [32]	America	325 (127)	1 y-Mortality 5 y-Mortality	L4-TPA T12-Dorsal muscle area	–	–
Montano-Loza (2014) [33]	America	248 (97)	Hospital stay Infections	L3-SMI SMA	112	M: < 53 cm^2^/m^2^ if BMI > 25 < 43 cm^2^/m^2^ if BMI < 25 W: < 41 cm^2^/m^2^ SMA < 41 HU if BMI < 24.9 < 33 if BMI > 25
Montano-Loza (2015) [34]	America	669 (291)	Mortality	L3-SMI	298	M: < 53 cm^2^/m^2^if BMI > 25 < 43 cm^2^/m^2^if BMI < 25 W: < 41 cm^2^/m^2^
Underwood (2015) [35]	America	348 (95)	Failure to rescue	TPA	–	–
Valero (2015) [36]	America	96 (67)	Surgical complications Mortality	L3-TPA TPV	44 by TPA 47 by TPV	M: < 680.4 mm^2^/m^2^ W: < 524.7 mm^2^/m^2^
Jeon (2015) [37]	Asia	145 (96)	Mortality	SMI	52 pre LT 66 post LT	M: < 7.7 cm^2^/m^2^if 20–50 years, < 6.6 cm^2^/m^2^if > 50 years W: 4.6 cm^2^/m^2^ if 20–50 years, < 4.4 cm^2^/m^2^ > 50 years
Carey (2016) [38]	America	396 (155)	Mortality	L3-SMI	178	M < 50 cm^2^/m^2^ W: < 39 cm^2^/m^2^
Itoh (2016) [39]	Asia	153 (153)	Surgical Outcome	SVR by TC	38	–
Van Vugt (2017) [40]	Europe	585 (193)	Mortality	L3-SMI	254	M: < 53 cm^2^/m^2^ if BMI > 25 < 43 cm^2^/m^2^ if BMI < 25 W: < 41 cm^2^/m^2^
Wada (2017) [41]	Asia	32 (2)	Respiratory complications	TPA TPV	16	TPA M: < 791.6 mm^2^/m^2^ W: < 488.8 mm^2^/m^2^ TPV M: < 149 cm^3^/m^2^ W: < 83.3 cm^3^/m^2^
Golse (2017) [42]	Europe	256 (102)	Intensive unite stay Complications mortality	PMA SMI	57	PMA M: < 1561 mm^2^ F < 1464 mm^2^
Chae (2018) [43]	Asia	408 (191)	Mortality Complications	Δ PMI	102	<—11.7%
Kim (2018) [44]	Asia	92 (92)	Recurrence	height-normalized Psoas muscle thickness	72	< 15.5 mm/m

*N* Number, *TPA* total psoas muscle area, *SMI* cross-sectional areas of skeletal muscle (cm^2^)/patient’s height (m^2^) , *TPMT/height* transversal psoas muscle thickness (mm) /height (m), *L4* fourth lumbar vertebra, *TPA* total psoas muscle area, *T12* twelfth thoracic vertebrae, *L3* third lumbar vertebra, *HU* transversal psoas muscle attenuation *TPV* total psoas muscle volume, *PMA* psoas muscle area, *PMI* psoas muscle index, *SVR* muscle mass-to-visceral fat area ratio

Sarcopenia was found to be associated also with the outcomes of LT once transplant was carried out. In particular, sarcopenia was associated with postoperative complications (including mortality), intensive care unit (ICU)/hospital stay and survival. According to a recent meta-analysis [46], there are only few studies focusing on the association between complications after LT and sarcopenia defined by either total psoas area (OR = 0.48 per increase in standard deviation of the area) [32] or volume (OR = 3.06) [36]. In the latter study, all severe complications (Clavien grade ≥ III, 23.4%) occurred in the sarcopenic group, although liver resections were also included in the same analysis. Others, instead, demonstrated a positive association between sarcopenia and rate of infection/sepsis [28], especially when of bacterial origin [33]. Among them, pneumonia was the most frequently described complication and responsible for longer ICU stay (due to more days of intubation) [29] and, in turn, for longer length of hospital stay and higher failure-to-rescue in such frail patients [35].

With regard to survival, a meta-analysis by Van Vugt et al. [46] showed a pooled HR of 1.84, which increased to 2.21 when only studies that measured psoas muscle area were included (heterogeneity from 60 to 49%). More recently, a study by Golse et al. [42] demonstrated that the 1- and 5-year survival rates were significantly poorer in the sarcopenic group than in the non-sarcopenic group (59% vs. 94% and 54% vs. 80%, *p* < 0.001). However, the largest contribution to survival came from the higher rate of 90-day or within 1-year mortality observed in the sarcopenic group [24, 33, 36]. Instead, when looking to HCC patients, since reduced skeletal muscle mass could lead to the decrease of certain cytokines (myokines and adipokines) and to the release of tumor necrosis factor (TNF)-α, such a combined effect was suggested to impact survival also by promoting tumor progression and recurrence after LT [44].

As regards living-donor liver transplantation (LDLT), a study by Kaido et al. [30] showed that the OS rate in patients with preoperative low skeletal muscle mass was lower than in those without (*p* < 0.001).

Another more recent study by Itoh et al. [39] including 153 patients undergoing LDLT showed that patients with low skeletal muscle mass-to-visceral fat area ratio (SVR) assessed by CT had a significantly poorer prognosis than those without both for recurrence-free (*p* = 0.01) and overall (*p* = 0.03) survival.

A more recent field of investigation is represented by the development of sarcopenia after LT, namely “de novo” sarcopenia, which is reported ranging between 15 and 25% of patients [37, 43]. However, it is more likely that sarcopenia was already present at the time of LT and then progressed after transplant. Many factors occurring after LT may be responsible for the decrease in lean body mass, including infections, renal dysfunction or lack of specific nutritional diets. Also, the use of immunosuppressive agents such as mTOR and calcineurin inhibitors may have an additional role [47]. In conclusion, sarcopenia can be predictive of poor outcomes in LT, thus offering the possibility to clinicians to improve the muscular status of these patients, especially those with high-grade sarcopenia and, therefore, at high risk of mortality both in the waiting list period and after transplant.

### Transarterial chemo- and radio-embolization

Trans-arterial chemoembolization (TACE) is the recommended treatment modality for asymptomatic, large or multifocal HCC patients, without macrovascular invasion or extrahepatic metastasis (intermediate HCC, BCLC stage B) [48]. Many independent factors influence the prognosis of patients treated with TACE and many of them are not tumour-related but are related to the individual patient characteristics. For example, it is well demonstrated and widely accepted that age, total bilirubin, alpha-fetoprotein (AFP) and ascites represent some important prognostic factors for patients treated with TACE [48–50]. Recently, the ability to perform non-invasive measurement of sarcopenia has facilitated the use of sarcopenia as a prognostic factor in many medical fields, such as in patients with liver diseases [12, 51, 52]. However, to date, the impact of sarcopenia on tumour response and OS in patients receiving TACE therapy has not been largely assessed (Table 4). Four studies had examined the role of skeletal muscle volume and its changes in HCC patients who received TACE [14, 53–55]. Two of these studies investigated patients undergoing TACE for primary liver cancer [53, 54], while the other two were conducted among patients who underwent TACE for both primary and secondary malignancies of the liver [14, 55].

**Table 4:** Studies assessing sarcopenia in patients undergoing TACE or TARE

Author (year)	Region	Technique	N. patients	Outcome	Methods for sarcopenia assessment	N. of sarcopenic patients	Cut off
Kobayashi (2018) [53]	Asia	TACE	102	Overall survival	L3 SMI ΔL3 SMI over 6 month	31 41	M: < 42 cm^2^/m^2^ W: < 38 cm^2^/m^2^ ΔL3 SMI < -4.6
Loosen (2019) [14]	Europe	TACE	56 (HCC = 46, Metastases = 10)	Treatment response Overall survival	PMI Δ PMI		< 11.8 mm/m^2^ < 13.39 mm/m^2^
Fujita (2019) [54]	Asia	TACE	179	Overall survival	PMI CPMI	80	M: < 6 cm^2^/m^2^ W: < 3.4 cm^2^/m^2^
Dodson (2019) [55]	America	TACE DEB TACE TARE	216 HCC = 109 Other = 107	Complication Overall survival	TPA	55	M: < 477 mm/m^2^ W: < 338 mm/m^2^
Faron (2020) [56]	Europe	TARE	58	Overall survival Progression free survival	FFMA	29	M < 3582 mm^2^ W < 2301 mm^2^

*CPMI* Changes in PMI per month during the TACE period, *BED TACE* drug-eluting bead TACE, *FFMA* derived fat-free muscle area, *HCC* hepatocellular carcinoma, *PMI* psoas muscle index, *SMI* skeletal muscle index, *TACE* trans-arterial chemoembolization, *TARE* trans-arterial radioembolization, *TPA* Total psoas area

These studies demonstrated that patients with progressive muscle hypotrophy after TACE had a significant decrease in OS. In Fujita et al. [54] series, the multivariate analysis showed that changes in psoas muscle index (PMI), a surrogate of sarcopenia, per month during the TACE period was significantly associated with poor OS (HR 1.884, *P* = 0.001, 95% CI 1.305–2.72, *p* = 0.001) [54]. Similarly, Kobayshi et al. [53] investigated the prognostic value of skeletal muscle loss (SML) stratified by cut-offs for sarcopenia and the rate of change in skeletal muscle mass over 6 months after TACE. The multivariate analysis revealed that SML was independently predictive of poor OS (HR, 1.675,95% CI 1.031–2.721; *P* = 0.037) with serum AFP ≥ 20 ng/mL (HR, 2.550; 95% CI 1.440–4.515; *p* = 0.001) and maximum tumour diameter ≥ 30 mm (HR, 1.925; 95% CI 1.166–3.179; *P* = 0.010) [53]. However, data regarding the prognostic role of pre-interventional sarcopenia remain controversial. Kobayashi et al. and Fujita et al. showed no significant association between muscle volume mass at baseline and clinical outcome [53, 54]. However, Loosen et al. and Dodson et al. [14, 55] similarly showed that pre-interventional sarcopenia was an independent predictor for an unfavorable outcome (respectively HR 2.876, 95% CI 1.044–7.922, *p =* 0.041 and HR 1.84, 95% CI 1.03–3.64, *P* = 0.04) [14, 55]. These data are similar to those of previously published series of patients with HCC treated with modalities different from TACE procedures [13, 18, 23, 57–60].

Interestingly, Loosen et al. [14] also demonstrated that pre-interventional sarcopenia did not correlate with treatment response to TACE (OR 0.704, 95% CI 0.494–1.003, *p* = 0.052) [14]. Besides, Dodson et al. [55] showed that baseline sarcopenia was not associated with the risk of periprocedural morbidity after TACE (OR 0.89, 95% CI 0.28–2.86, *p* = 0.84) [55]. These data probably establish that the prognostic impact of sarcopenia on TACE depends on its role over the general clinical conditions of cirrhotic patients with HCC and not directly on its role on the local efficacy of TACE (evaluated by tumour response or periprocedural morbidity). However, these preliminary results need to be confirmed with more robust data before introducing these concepts in daily clinical practice.

If there is little scientific evidence for TACE, the role of sarcopenia in the field of trans-arterial radioembolization (TARE) has been even less investigated. To the best of our knowledge, only one recent study investigated the impact of sarcopenia in HCC patients treated with TARE [56]. In particular, Faron et al. [56] evaluated the value fat-free muscle area (FFMA) as a marker of sarcopenia to predict clinical outcomes in patients receiving TARE for treatment of unresectable HCC (14). FFMA as a measure of lean muscle mass was identified as an independent prognostic factor of OS (HR 2.675, 95% CI, 1.255–5.702, *P* = 0.011) and seems to provide significant prognostic information on the OS in patients receiving TARE for the treatment of HCC [56].

In conclusion, the assessment of sarcopenia could provide additional prognostic information beyond established biomarkers and, moreover, may also help to further stratify the patients in order to optimize the selection criteria for receiving TACE or TARE for treatment of unresectable HCC. However, most of these data are derived from retrospective studies involving a relatively small number of patients. Future prospective studies with larger sample size and longer observation are required to provide more robust evidence about the prognostic role of sarcopenia in HCC patients receiving trans-arterial therapies such as TACE and TARE and to reveal whether the prevention of skeletal muscle depletion might contribute to improving clinical outcomes.

### Systemic therapies

The assessment of sarcopenia is a useful tool in oncologic settings, particularly in patients with advanced oncologic disease, since they are exposed to several cancer-specific and non-cancer-specific factors causing decrease in muscle mass and muscle dysfunction [61]. As cancer progresses to unresectable or metastatic stage, possible treatments are often limited to systemic therapy and the patients present higher prevalence of sarcopenia [62]. In many different types of cancers, sarcopenia has been shown to be a prognostic factor for disease progression, OS, response to treatments, poor performance status and toxicity caused by chemotherapy [4, 63–65]. Similarly, in the past decade, some papers established the prognostic role of sarcopenia in cirrhotic patients who underwent systemic therapy for hepatocellular carcinoma, even if they mostly focused on Sorafenib-based regimens (Table 5). Sorafenib is the first orally active multi-kinase inhibitor that has been confirmed to be efficacious in patients with advanced HCC [73], representing today the standard first-line treatment [74], however, it may cause many different side effects, such as fatigue and diarrhea, up to hand-foot syndrome and liver dysfunction, which may lead to dose reduction or treatment interruption [75, 76]. First data about sarcopenia in Sorafenib regimen for advanced HCC were described in 2012, when Mir et al. [16] established that sarcopenia was independently correlated with the occurrence of early dose-limiting Sorafenib toxicities (DLTs) in patients who have advanced HCC with Child–Pugh A liver cirrhosis, also describing a significative prevalence of grade 3 diarrhea in sarcopenic patients. Few years later, in 2015 a Japanese study [66] first provided the prognostic role of sarcopenia in patients undergoing Sorafenib for HCC, measuring by CT-scans the L3 SMI. L3-SMI was identified as independent prognostic factors in HCC patients treated with Sorafenib (*p* = 0.020), with an OS significantly shorter in patients with L3 SMI <  = 39.2 cm^2^/m^2^ (*p* = 0.003). Nevertheless, in this cohort of 40 patients with HCC, L3 SMI did not appear to be a significant risk factor for dose reduction or discontinuation of Sorafenib.

**Table 5:** Studies assessing sarcopenia in patients undergoing Sorafenib therapy

Author (year)	Region	N. patients	Outcome	Methods	N. sarcopenic	Cut off
Mir (2012) [16]	Europe	40	Dose limiting toxicities	L3-SMI	11	M: < 55.4 cm^2^/m^2^ F: < 38.9 cm^2^/m^2^
Imai (2015) [66]	Asia	40	Mortality	L3-SMI	15	< 29.2 cm^2^/m^2^
Nishikawa (2017) [67]	Asia	232	OS Progression-free survival	L3-SMI	151	M: < 36.2 cm^2^/m^2^ F: < 29.6 cm^2^/m^2^
Hiraoka (2017) [68]	Asia	93	OS Time to progression Time on treatment	PSI	20	M: < 4.24 cm^2^/m^2^ F: < 2.50 cm^2^/m^2^
Yamashima (2017) [69]	Asia	40	OS Progression free survival	ΔTPMT/height	–	0.59 mm/m
Takada (2018) [70]	Asia	214	OS	L3-SMI	123	M: < 42 cm^2^/m^2^ F: < 38 cm^2^/m^2^
Antonelli (2018) [15]	Europe	96	OS Time on treatment	L3-SMI	47	M: < 53 cm^2^/m^2^ if BMI > 25 < 43 cm^2^/m^2^ if BMI < 25 W: < 41 cm^2^/m^2^
Saeki (2018) [71]	Asia	100	OS	L3-SMI VFA	46	M: < 42 cm^2^/m^2^ F: < 38 cm^2^/m^2^ VFA > 100 cm^2^
Imai (2019) [72]	Asia	61	OS	L3-SMI ΔVFMI, ΔSFMI, ΔL3-SMI	25 before sorafenib	M: < 42 cm^2^/m^2^ F: < 38 cm^2^/m^2^ ΔL3SMI > -5.73 cm^2^/m^2^/120 days ΔSFMI > -5.33 cm^2^/m^2^/120 days ∆VFMI > − 3.95 cm^2^/m^2^/120 days

*N* Number, *L3* third lumbar vertebra, *SMI* cross-sectional areas of skeletal muscle (cm^2^)/patient’s height (m^2^), *OS* Overall Survival, *PSI* psoas muscle area at level of middle of third lumbar vertebra (cm^2^) / height (m^2^), *TPMT/height* Transversal psoas muscle thickness (mm)/ height (m), *VFA* Visceral fat area, *VFMI* visceral fat mass index, *SFMI* subcutaneous fat mass index

Successively, a larger retrospective study [67] conducted on 232 patients with unresectable HCC established a significantly reduced OS after Sorafenib treatment in sarcopenic patients versus non-sarcopenic ones (174 vs. 454 days, *p* < 0.0001), confirming sarcopenia assessed by L3 SMI as an independent predictor factor of OS (HR 0.365). Sarcopenia-group presented as well significantly lower objective response rate (*p* = 0.0146) and disease control rate (*p* = 0.0151), compared with non-sarcopenia group. Similarly, Takada et al. [70] evaluated pre-sarcopenia (established according to the standard proposed by Japan Society of Hepatology [77] with L3 SMI in CT-scans < 42 cm^2^/m^2^ in males and 38 cm2/m2 in females) and its role in 214 patients with advanced HCC treated with Sorafenib. They found that OS in patients with pre-sarcopenia tended to be worse than that of the control group, even if not significantly (252 vs. 284 days, *p* = 0.16), with bigger differences after stratification of the study cohort by prognostic factors. In fact, in the subgroup who had three or more negative prognostic factors, the presence of pre-sarcopenia did not correlate with prognosis, while in the subgroup with two or less prognostic factors, the OS in pre-sarcopenic patients was significantly lower (HR 1.6, *p* = 0.047); even in this study, no association between pre-sarcopenia and sorafenib treatment duration or dose reduction was observed. Differently, a European multicentric retrospective study [15] in a cohort of patients who underwent Sorafenib found that sarcopenia (assessed by L3 SMI) was significantly associated with patients OS (63 vs. 32 weeks, HR 1.69, *p* = 0.02), with a reduced duration of treatment among sarcopenic patients (25.8 vs. 12.3 weeks, HR 1.75, *p* = 0.0044) and with sorafenib-related toxicity (adverse events grade 3 and 4, 62% vs. 40%, *p* = 0.04).

The prognostic impact of body composition in advanced HCC patients treated with Sorafenib was also investigated by Saeki et al. [71] which analysed the pre-treatment depletion muscle mass with L3-SMI and visceral fat area (VFA) at umbilical level using CT images. They found that the absence of muscle depletion (HR = 0.498, *p* = 0.006), and a value of VFA >  = 100 cm^2^ (HR = 0.556, *p* = 0.031) were significant factors for long-term survival, higher disease control rate (*p* = 0.012) and longer duration of treatment, with a higher total amount of Sorafenib administered.

New interesting data are now available by the longitudinal and dynamic evaluation of sarcopenia changes before and after Sorafenib treatment of advanced HCC patients for predicting the patients’ outcomes. As example, Hiraoka et al. [68] showed that the value of psoas muscle area index (PSI) was significantly reduced at follow-up CT-scans, at 8–12 weeks after starting Sorafenib treatment. A similar effect is likely due to the antiangiogenic characteristics of Sorafenib, which appears to directly inhibit protein synthesis, as already observed in other oncologic settings [78]. According to Yamashima et al. [69], the variation of skeletal muscle thickness, before and after 1–3 months of Sorafenib treatment, resulted to be an independent factor for OS (*p* = 0.0439, HR = 1.99271). Differently from the studies above, this group established skeletal loss thickness assessing Transverse Psoas Muscle Thickness over height (TPMT/height), through CT-images at the level of the umbilicus.

Finally, a recent, more comprehensive study was conducted by Imai et al. [72] assessing visceral fat mass index (VFMI), subcutaneous fat mass index (SFMI), L3 SMI and relative changes of such indexes (ΔVFMI, ΔSFMI and ΔL3SMI) before and after Sorafenib treatment (120 days). Patients within the 20th percentiles cut-offs for ΔL3SMI, ΔSFMI and ΔVFMI were classified into a rapid depletion group. Confirming the above literature, baseline-sarcopenic patients showed reduced survival than those without (*p* = 0.0157). In addition, rapid depletion group, according to ΔSFMI and ΔL3SMI, showed a significantly poorer survival too (respectively *p* = 0.0101 and *p* = 0.0027). Multivariate analysis confirmed that the presence of sarcopenia (HR 2.453, *p* = 0.007), ΔSFMI >  = 5.33 (HR 4.109, *p* =  < 0.001) and ΔL3SMI >  = 5.73 (HR 4.010, *p* < 0.001) were independent predictors of survival.

As far as other systemic therapy for advanced HCC, to our knowledge there is poor evidence regarding the prognostic role of muscle mass, especially considering the recently approved Regorafenib or other promising molecules, such as metronomic Capecitabine, Lenvatinib, Nivolumab, Cabozantinib or Ramucirumab [79]. Less recent studies [80, 81] have been published regarding the combination of gemcitabine and oxaliplatin (GEMOX regimen) as second-line treatment in advanced HCC,even in these cohorts of patients, a significantly shorter OS was described in sarcopenic patients. Lately, a new interest has been growing about Lenvatinib, which, unlike Sorafenib, appears to induce a minimum, non-significant, muscle loss, even after 24 months of treatment for unresectable HCC: this may be correlated with the positive clinical response and its low toxicity [82]. However, the initial expectations were disappointed since a multicentric study [83] on 152 unresectable-HCC patients showed a relative reduction in muscle volume at 4 and 12 weeks after starting Lenvatinib in 35.3% of subjects, even if these findings have not yet been related to patients outcome and HCC response. In conclusion, sarcopenia has been assessed as a well-recognized predictive factor of poor prognosis in patients treated with Sorafenib for advanced HCC, both at baseline and as a change through the treatment time. Its role in predicting HCC response or chemotherapy toxicities tolerability needs to be deepened since studies published showed conflicting data. In the same way, new evidences needs to be produced about sarcopenia and new agents’ therapy, in order to better target systemic treatments on HCC patients.

## Future perspectives

### Improving sarcopenia in liver cirrhosis

By the way sarcopenia has an important role in defining the prognosis of cirrhotic patients, several authors tried to find a strategy to improve this condition in these patients. Most literature is focused on the effect of physical exercise in cirrhotic patients [84–87], but there is no specific evidence that exercise can reverse sarcopenia in this setting. However, it seems reasonable to suggest that physical therapy, when tolerated, may help prevent further loss of muscle mass [88–90]. The effects of exercise in sarcopenic patients might be explained by stimulation of mTOR signaling, inhibition of muscle apoptosis by decreasing local TNF-α levels, stimulation of mitochondrial oxidative capacity and increased blood flow to the skeletal muscle [91, 92]. An example of physical activity recommended is walking 30–40 min three or four times per week and lifting light weights such as hand weights two to three times per week [88]. A study by Aemann et al. [93] evaluated the efficacy of progressive resistance exercise in cirrhotic patients compared to a control group. They found that the exercise group increased their quadriceps cross-sectional area of 10%, greater gain than that of the control group (*p* < 0.01) [93]. Other studies evaluated the impact of nutritional supplementation in cirrhotic patients. In a study by Ohara et al. in 2018 [94] the authors evaluated the supplementation of L-Carnitine, comparing the PMI before and after supplementation. The Δ PMI/ month value (Δ PMI/month (%) = ([psoas muscle area on the second CT scan – psoas muscle area on the initial CT scan]/psoas muscle area on the initial CT scan) × 100/interval between CT scans (m)) was 0.27% in the group with the supplementation of L-Carinitine and − 1.24% in the control group, with a significant difference (*p* < 0.01) both in males and females. Another similar study [95] evaluated the metabolic and molecular response to branched-chain amino acids (BCAA) enriched with leucine in six well-compensated alcoholic cirrhosis compared to controls; the authors found that a large dose of supplemental leucine was able to overcome the skeletal muscle anabolic resistance in cirrhosis, better than with BCAA alone.

Finally, Hiraoka et al. [96] analysed the combined effect of BCAA supplementation and walking exercise to prevent sarcopenia in cirrhotic patients. The alimentary supplementation consisted of a late-evening snack (protein 13.5 g, including L-leucine 1922.5 mg, 210 kcal/day) and additional 2000 steps/day to the normal routine for 3 months. The ratio of muscle volume changed from 1.0 to 1.013 (*p* < 0.01), as observed using the bioelectrical impedance analysis (BIA) method, and also leg and handgrip strength changed significantly (*p* < 0.01) [96].

### Improving sarcopenia before and after HCC treatment

As regards sarcopenia treatment in patients with HCC, scarce evidence is to date available. Koya et al. [97] evaluated the effects of in-hospital therapeutic exercise on liver function and skeletal muscle mass after HCC treatment (85% TACE) in patients with chronic liver diseases. They used a combination of a 20-min lasting exercise to be performed already during the hospitalization starting from the subsequent day of the HCC treatment for all patients. The patient’s body weight decreased significantly during hospitalization together with the skeletal muscle mass measured with BIA, except for the right arm. Notably, in the patients previously under treatment with BCAA the decrease of muscle mass was lower than that of other patients who only made exercise ( – 0.5 kg vs  – 1.1 kg) [97]. However, the exercise improved physical ability without worsening liver function.

The same authors [98] in a subsequent study including patients with HCC who underwent TACE identified two groups: patients performing exercise (*n* = 102) and controls (*n* = 107). The authors evaluated SMI and PMI before TACE and 50 days after, after treatment, the ΔPMI and ΔSMI were significantly higher in the exercise group than in the control group; this discrepancy between the two studies may be explained by the methodological differences in evaluation of SMI (BIA vs CT scan images of the abdominal cross-sectional area at the level of L3) [98].

A similar study [99] evaluated the effect of cancer rehabilitation (CR) on the prognosis of patients with HCC who underwent TACE. CR, a new multidisciplinary intervention for cancer patients, consists of nutritional and physical therapy. Patients were classified into the CR (*n* = 85) and control (*n* = 67) groups and they evaluate the change in SMI. After treatment with TACE, ΔSMI was significantly higher in the CR group than in the control group (*p* = 0.02). On the other hand, there was no significant difference between the CR and control groups regarding the ΔSMI in male patients [99]. Other studies were focused on patients belonging to the LT setting. The cornerstone of these studies [100] consisted in a pre-transplant nutritional supplementation. Enteral supplementation improved parameters of nutritional status pretransplant and mid-arm circumference, mid-arm muscle circumference and grip strength, but it had no effect on the outcomes of LT. Finally, other authors [101, 102] investigated the role of physical exercise in cirrhotic patients on LT waiting list on hospital stay, 1-year mortality and morbidity after LT and related adverse events, but, however, no randomized clinical trial evaluated its impact in reducing sarcopenia or in outcome changes after LT. In conclusion, nutritional supplementation and physical exercise seem to be a reasonable intervention to reduce sarcopenia in cirrhotic patients in order to prevent complications in the eventuality of HCC occurrence and treatment. Sarcopenia treatment for improving the nutritional status of the patients seems to be not feasible between HCC occurrence and treatment due to the slowness of the recovery process, as also witnessed by the lack of studies in this timeframe. On the other hand, limited data are available on interventions made after HCC treatment in order to improve survival. Thus, further prospective, larger, well-designed and with standardized parameters studies are need in order to evaluate the effect of interventions such as nutritional supplementations and physical activity in patients who undergone HCC treatments.

#### Conclusions

Several studies evaluated the predictive role of sarcopenia assessment before HCC treatments. Steady evidence is almost available on the prediction of survival of patients undergoing liver resection, transplantation and Sorafenib. However, most of the evidence came from eastern studies using different methods to assess sarcopenia with different cut-offs. Further studies are needed to clarify the role of sarcopenia in other HCC treatment settings and to address a possible utility as an additional staging tool for identifying the most appropriate treatment. Besides, interventional studies aiming at increasing the skeletal muscle mass for reducing complications and increasing the survival of a given HCC treatment are needed.

## Abbreviations

HCC: Hepatocellular carcinoma
BCLC: Barcelona clinic liver cancer
ECOG: Eastern cooperative group
OS: Overall survival
ICGR-15: Indocyanine green retention test at 15 min
BMI: Body mass index
SMI: Skeletal muscle index
HR: Hazard ratio
CI: Confidence interval
TFLV: Total functional liver volume
TPA: Total psoas area
ISGLS: International study group of liver surgery grading
OR: Odds ratio
PHLF: Post hepatectomy liver failure
RFA: Radiofrequency ablation
PMI: Psoas muscle index
CT: Computed tomography scan
ROC: Receiver operating characteristics
AUROC: Area under receiver operating characteristics curve
ESLD: End-stage liver disease
LT: Liver transplantation
TMPT: Transverse psoas muscle thickness
MELD: Model for end-stage liver disease
ICU: Intensive care unit
TNF: Tumor necrosis factor
TACE: Trans-arterial chemoembolization
AFP: Alpha-fetoprotein
SML: Skeletal muscle loss
TARE: Trans-arterial radioembolization
FFMA: Fat-free muscle area
VFA: Visceral fat area
PSI: Psoas muscle area index
VFMI: Visceral fat mass index
SFMI: Subcutaneous fat mass index
BCAA: Branched chain amino acids
BIA: Bioelectrical impedance analysis
CR: Cancer rehabilitation
